# Addressing health inequalities in Europe: key messages from the Joint Action Health Equity Europe (JAHEE)

**DOI:** 10.1186/s13690-023-01086-3

**Published:** 2023-05-11

**Authors:** Raffaella Bucciardini, Pi Zetterquist, Tuulia Rotko, Vania Putatti, Benedetta Mattioli, Paola De Castro, Federica Napolitani, Anna Maria Giammarioli, Bernadette N. Kumar, Charlott Nordström, Christina Plantz, Yvette Shajanian Zarneh, Gabriella Olsson, Malin Ahrne, Katri Kilpeläinen, Daniel Lopez-Acuña, Apostolos Vantarakis, Michele Marra, Cecilia Nessi, Giuseppe Costa

**Affiliations:** 1 Istituto Superiore Di Sanità, Rome, Italy; 2 Public Health Agency of Sweden, Stockholm, Sweden; 3 Finnish Institute for Health and Welfare, Helsinki, Finland; 4 EuroHealthNet, Brussels, Belgium; 5 Norwegian Institute of Public Health, Oslo, Norway; 6 Federal Centre for Health Education, Cologne, Germany; 7 Andalusian School of Public Health, Granada, Spain; 8 Medical School, University of Patras, Patras, Greece; 9 Epidemiology Unit, ALSTO3, Piedmont Region, Turin, Italy; 10 Eclectica, Turin, Italy; 11 Dept Clinical and Biological Sciences, Turin University, Turin, Italy

**Keywords:** Health inequalities, Public health, Global health, Governance, Health systems

## Abstract

Health inequalities within and between Member States of the European Union are widely recognized as a public health problem as they determine a significant share of potentially avoidable mortality and morbidity. After years of growing awareness and increasing action taken, a large gap still exists across Europe in terms of policy responses and governance. With the aim to contribute to achieve greater equity in health outcomes, in 2018 a new Joint Action, JAHEE, (Joint Action Health Equity Europe) was funded by the third EU Health Programme, with the main goal of strengthening cooperation between participating countries and of implementing concrete actions to reduce health inequalities. The partnership led by Italy counted 24 countries, conducting actions in five policy domains: monitoring, governance, healthy living environments, health systems and migration, following a three-step implementation approach. Firstly, specific Policy Frameworks for Action (PFA) collecting the available evidence on what practice should be done in each domain were developed. Second, different Country Assessments (CAs) were completed to check the country’s adherence to the recommended practice in each domain. The gap between the expected policy response (PFA) and the present policy response (CA) guided the choice of concrete actions to be implemented in JAHEE, many of which are continuing even after the end of JA. Final recommendations based on the best results achieved during JAHEE were elaborated and agreed jointly with the representatives of the involved Ministries of Health. The JAHEE initiative represented an important opportunity for the participating countries to work jointly, and the results show that almost all have increased their level of action and strengthened their capacities to address health inequalities.

## Background

Health inequalities can be defined as avoidable and unjust differences in the health status attributable to the social determinants of health which are the conditions in which people are born, live and work [1]. Health inequalities within and between Member States (MS) of the European Union (EU) are widely recognized as a public health problem as they determine a significant share of potentially avoidable mortality and morbidity [2–6]. The 2008 report of the WHO Commission on Social Determinants of Health (CSDH), *Closing the gap in a generation,* concluded that “social injustice is killing people on a grand scale” [1]. The CSDH report provided a comprehensive synthesis of knowledge and evidences on health inequalities across Europe as well as a set of recommendations to develop wide and integrated policies to contrast them. It is considered by many countries as a reference text on health inequalities and social determinants of health. The CSDH report was followed by the Rio Political Declaration on Social Determinants of Health in 2011 which was adopted by more than 100 countries [7]. In 2013 a review of health inequalities across the 53 countries of the European Region, the *WHO Review of the Social Determinants and the Health Divide* [8], further underlined the need to act on social determinants of health with the final recommendation to “*do something, do more, do better*”. To address these inequalities in health and in policy response, in 2011 a 3-year Joint Action (JA) on health inequalities called “Equity Action” was funded by EU Commission to encourage collaboration and sharing of knowledge between MSs with the aim of elaborating and testing new tools and methods for health inequalities impact assessment and audit [9]. However, despite the new tools, there is still a great gap throughout Europe in terms of concrete actions to improve health equity in the field, including health leadership and governance at national and local level. This scenario was only bound to worsen in the future as new challenges arise, such as the increase of migration flows caused by humanitarian and economic crises across the globe. The outbreak of the COVID-19 pandemic further accentuated these challenges and their link to the unsustainable cost of health and to social inequalities in our societies [9].

In this context a new EU JA, JAHEE (Joint Action Health Equity Europe), began in 2018 with the main goal of strengthening a cooperative approach to improve countries capacities to develop policies and implement action to reduce health inequalities [10]. This paper aims at providing a description of the activities carried out under JAHEE. Upon a brief contextualisation, we describe the structure of the project as well as the main actions put in place to foster health and equity across the EU. The article concludes with a brief overview of the final recommendations resulted from the project activities.

## Methods

JAHEE was an EU initiative funded by the third EU Health Programme 2014–2020. Initially foreseen for the duration of three years, it was extended for additional 6 months due to the COVID-19 pandemic crisis (June 1, 2018 – November 30, 2021).

**Fig. 1:**
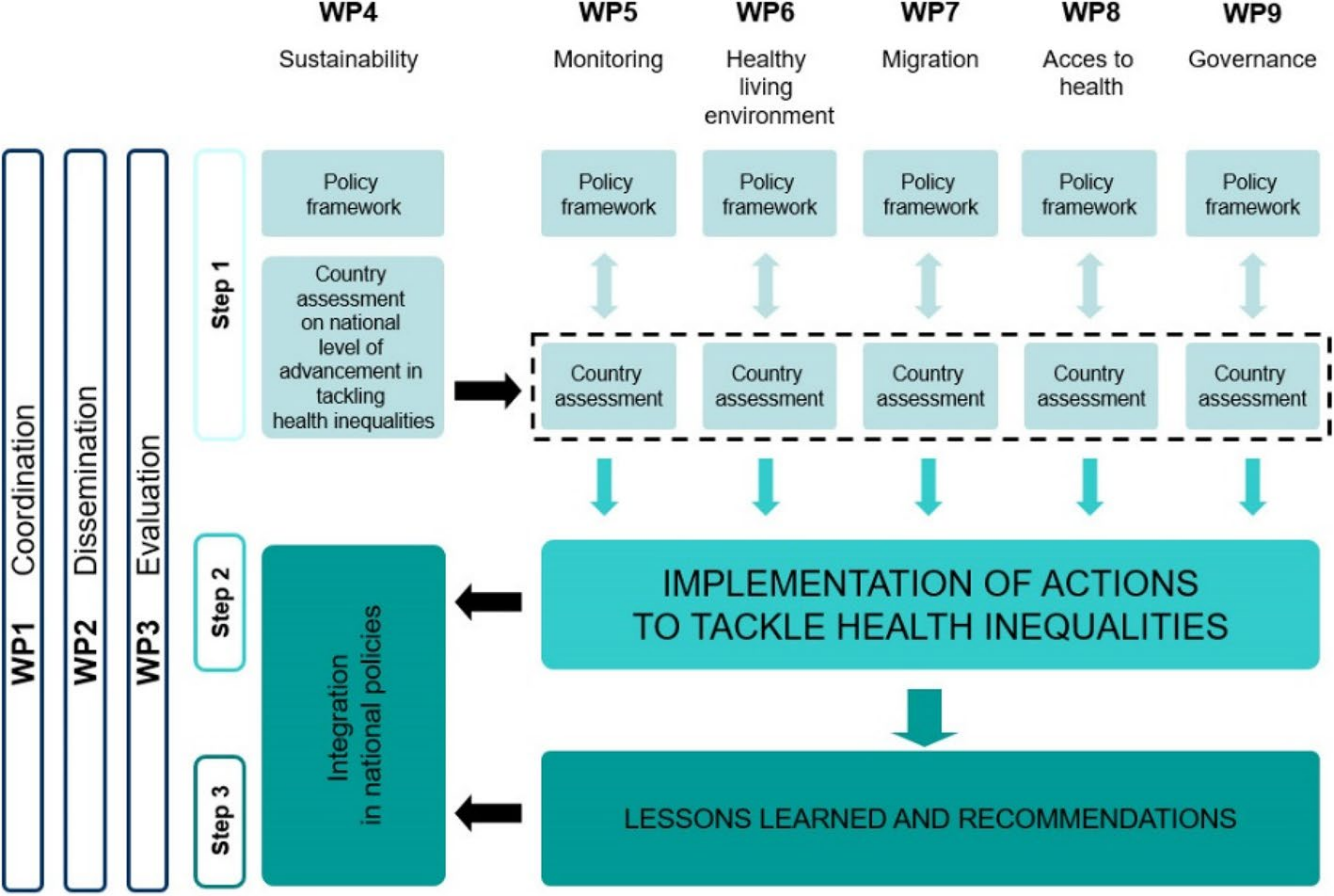
Visual scheme of the JAHEE structure

The partnership, led by Italy, counted 24 countries (21 EU MSs: Belgium, Bulgaria, Croatia, Cyprus, Czech Republic, Estonia, Finland, France, Germany, Greece, Italy, Lithuania, Netherlands, Poland, Portugal, Romania, Slovakia, Slovenia, Spain, Sweden, United Kingdom plus Norway, Serbia and Bosnia and Herzegovina). JAHEE’s activities were carried out through nine Work Packages (WP) (Table 1). The participation of the partners was mandatory in WP1 (coordination), WP2 (dissemination), WP3 (evaluation) and WP4 (integration in national policies and sustainability), while it was on a voluntary basis in the five policy domains such as WP5 (monitoring), WP6 (healthy living environments), WP7 (migration and health), WP8 (improve access to health and related social services for those left behind), WP9 (governance and health). JAHEE followed a three-step implementation approach (Fig. 1):

1. In the first phase, the five thematic WPs were responsible for collecting the best available knowledge about the effectiveness of the practices aimed at reducing health inequalities in the respective domains, resulting in five Policy Frameworks for Action (PFA) in *monitoring, governance, living environment, health system and migration*, under the coordination of a general PFA (by WP4) summarising the available evidence on the mechanisms generating health inequalities as potential entry point for action. At the same time at country level, a general (WP4) and specific (WP5-WP9) Country Assessments (CAs) were completed with the aim of acquiring evidence on the degree and capacity of inclusion of practices recommended in PFAs. Comparing the standard capacity in policy response (expected according to PFA) to the actual situation (taken in the snapshot of the CA) each partner was able to recognize its own needs and priorities for improvement;
2. In the second phase, the information collected in each CAs and in the six-PFAs was used to guide individual partners in choosing actions to be implemented in the WPs also taking into account constraints and opportunities from the context including the incipient pandemic.
3. Finally, recommendations based on the best results achieved during JAHEE were produced.

**Table 1:** JAHEE Work Packages

**WP 1—Management of the Action (Coordination)** Actions undertaken to manage the Joint Action and to make sure that it is implemented as planned
**WP 2 – Dissemination** Actions undertaken to ensure that the results and deliverables of the Joint Action are made available to the target groups
**WP 3—Evaluation** Actions undertaken to verify if the Joint Action is being implemented as planned and reaches the objectives
**WP 4—Integration in National Policies and Sustainability** Actions undertaken to ensure that the Joint Action implementing efforts of all WPs frame into a bigger picture of integration and sustainability for strengthening national efforts to tackle health inequalities
**WP 5 – Monitoring** Actions undertaken to support participating countries to develop monitoring of health inequalities, adapted to the national context, and sustainable over time and to develop and use health inequalities indicators for health policy evaluation and prioritization
**WP 6 – Healthy Living Environments** Actions undertaken to support participating countries identifying national strategies and policies and models of good practice to better understand assets and impacts of living environments on healthy life styles, risk and resilience factors; develop implementation guidelines for healthy urban planning, and advocacy guidance for decision makers and stakeholders
**WP 7—Migration and Health** Actions undertaken to advance migrants health by effective and comprehensive health system responses for migrants; reduce fear and misconceptions related to migrants’ health that contribute to health inequalities for migrants
**WP 8—Improving Access to Health and Related Social Services for those Left Behind** Actions undertaken to reduce HI in access to health and social services, through the formulation of regional, national and local strategies, policies and programs, and to build participating countries capacity to promote social cohesion through the reduction of health inequalities in the access to health services
**WP 9—Health and Equity in All Policies – Governance** Actions undertaken to strengthen participating countries capacity, abilities and commitment to develop and implement effective and concrete policy actions to tackle health inequalities from national to local levels and building multisectoral collaboration

The project evaluation (including the evaluation on actions implemented) was based on systematic and continuous monitoring of processes, outputs and outcomes indicators aimed to guarantee the achievement of the planned objectives and the identification of the lessons learned for future programs. The project was evaluated internally by WP3 and externally by an by an independent evaluation conducted by the Center for Global Health Inequality Research (CHAIN).

JAHEE resulted in 27 deliverables and 45 milestones collected in a number of WP-specific documents and reports. Most of them are public and available in the JAHEE website [11]. This paper aims at presenting the main actions that JAHEE partnership designed and started in the five policy domains.

## Results

Seventy-six actions started during JAHEE and some of them are continuing beyond the end of project. Twelve countries participated in WP5-Monitoring (Cyprus, Finland, Germany, Italy, Lithuania, Netherlands, Poland, Romania, Serbia, Slovenia, Spain, Sweden) and fifteen actions were initiated and implemented in partner countries with the aim to improve their current health inequality monitoring system (HIMS). Out of the 15 actions foreseen under WP5, five focussed on strategy development, seven on methodology, three on improving communication or dissemination. Shown in the box are some actions taken by the JAHEE partners to invest in monitoring health inequalities:

**Table:** 

Slovenia and Sweden both used their involvement in JAHEE as an opportunity to set up a sustainable HIMS) at national level -In Slovenia, the National Institute of Public Health prepared a strategic plan detailing a systematic approach to long-term monitoring of HI complete with goals, methodology, a pragmatic set of indicators, and an evaluation plan. As part of the plan, they also systematically identify all relevant stakeholders and defined JAHEE [HP-JA-2017] [801600] • 9 channels of communication, to establish a strong network of people and organisations that can help to facilitate actions to tackle HI. -The Public Health Agency of Sweden (FOHM) was commissioned by the government to develop a HIMS that is aligned across levels of governance in an Health in All Policies (HiAP) approach. The proposal includes the content and the format of the system and will be further developed in collaboration with regional and local actors. The information collected will be key to assessing progress towards a public health target adopted by the government, to close avoidable HI gaps in a generation.

**Table:** 

Spain set up a city-based Health Inequalities Monitoring System, that could be scaled to other cities. The aim of the action was to set up a system to monitor health inequalities in Barcelona, which would, down the line, form part of the information system of the Public Health Observatory in Barcelona. A group of experts from the Agència de Salut Pública de Barcelona (Public Health Agency of Barcelona-ASPB) reviewed the literature and designed a system to monitor health inequalities in the city. Eight indicators were chosen covering both health determinants (income), health-related behaviours and use of health services (overweight and obesity, odontology visits), and health outcomes (poor self-perceived health, poor mental health, life expectancy, teenage pregnancy, and COVID-19 incidence).

**Table:** 

Romania investigated, as part of JAHEE, the possibility of establishing a system to monitor inequalities in reproductive health in their country, to support political decisions with evidence for implementing a general health inequality monitoring system. To this effect, the action focused on: developing a profile of the reproductive health in Romania, defining a set of indicatorsthat could be collected periodically within the annual National Programme on Mother and Child Health, and drafting a proposal of ministerial order with respect to the implementation of inequality monitoring system in the field of reproductive health in Romania. A feasibility analysis revealed that it is possible to start by piloting a short set of monitoring indicators in the field of reproductive health, in the first year.	

Thirteen countries participated in WP6-Healthy living environments *(Czech Republic, Cyprus, Germany, Greece, Italy, Netherlands,* Poland*, Portugal, Romania, Serbia, Slovenia, Spain, Sweden)* and fourteen concrete actions were designed and implemented by JAHEE countries. Most of the actions were implemented at local level and included capacity building and training activities. Most of the actions addressed environmental risks, social cohesion and social capital, physical activity and integrated approaches. Shown in the box are some actions taken by the JAHEE partners to strengthen municipal capacities.

**Table:** 

As part of JAHEE, the Public Health Agency in Sweden developed a Guide for Healthy Urban Planning and Development that links to both national and international frameworks, focusing on how our built environment can contribute to health equity. The guide contains information and brief guidance on how to work for health equity at regional and local level through urban planning, e.g., mobility, housing and green structure. The guide demonstrates how, at national level, there are many different goals, strategies and policies that are relevant for health equity. The structure of Agenda 2030 provides a good illustration of how all sectors can contribute to health equity and sustainability.

**Table:** 

As part of JAHEE, the Netherlands (Pharos) supported intersectoral action on health equity in Dutch municipalities. The aim of the action was to stimulate health equity in all policies, by strengthening intersectoral cooperation JAHEE [HP-JA-2017] [801600] • 15 between the health, social and urban planning sector. Three municipalities participated in this action (Nunspeet, Maastricht and Utrecht) to: frame health equity within a physical environment setting; implement health equity within a neighbourhood renovation process and share knowledge to convince urban planners to take health equity into account. In Nunspeet, a joint vision was formed to combine health and spatial issues, based on the PPP (people, planet, profit) vision, whilst in Maastricht, the “positive health” vision (a broad concept of health and its dimensions) was used as a joint approach for deprived areas. Lastly, in Utrecht, the knowledge shared by the scientists was concisely summarised into one-liners and easy quotes, which were instantly taken up by health advisors.

**Table:** 

As part of JAHEE, the Basque Country in Spain organised a capacity building process aimed at making local politicians aware of HiAP and equity in health. The action consisted of training sessions and active mapping activities targeting local technical and political personnel, through a community participative process. It required specific mandatory prerequisites that had to be fulfilled by the municipality (a budget for Health Promotion and Equity in Health, a Health Promotion Reference professional designated in the municipality and the inclusion of Health Promotion in the political agenda). The action will serve as a model to be replicated in other municipalities.

**Table:** 

As part of JAHEE, the National Institute of Public Health in the Czech Republic implemented ‘Parks in Motion ‘, as a long-term initiative to offer physical activity for all age groups and completely free of charge. The project encourages children, adults and seniors to exercise regularly in the city park under the guidance of qualified trainers. Implementing the programme required the cooperation of various entities at several levels. The key findings of this initiative were that successful health promotion at the local level requires effective support at the national level and sufficient resources in terms of capacity building.

**Table:** 

A program to improve the personal hygiene skills of children and their parents, including dental hygiene, was implemented as part of JAHEE by the Ministry of Health, the Ministry of Education as well as several Local Health Units in the 6th Health Region in Cyprus. It involved 152 kindergartens and more than 6,000 children and their parents, encouraging and enabling the creation of a healthier environment and healthier life skills in kindergartens as well as at home. This program emphasizes the importance of the multi-level involvement of all stakeholders in health promotion and the need for actions outside health system in order to reduce inequalities and promote health.	

Ten countries participated in WP7-Migration and Health *(Czech Republic, Finland, Greece, Italy, Norway, Portugal, Serbia, Spain, Sweden, and Wales).* Fourteen actions were implemented in six priority areas: 1. data and research; 2. governance; 3. intersectoral action on social determinants of health; 4. access to health services; 5. quality of health services; 6. attention to vulnerable groups. Shown in the box are some actions taken by the JAHEE partners to mainstream migration into all aspects of health policy:

**Table:** 

In the context of JAHEE, the National Institute for Health and Welfare in Finland brought forward a specific action to monitor and share information of health, wellbeing, and access to care among foreign born population living in Finland, as well as to design a systematic long-term monitoring tool to evaluate health inequality among this group. The action supported increased collaboration with extensive existing networks to achieve a very successful data collection among the foreign-born population living in Finland, which was needed both nationally and regionally. Efforts were additionally made to ensure that information will be collected regularly from those of foreign origin for the purposes of health inequality monitoring at both national and regional levels. Moreover, the widespread sharing of such evidence-based information in different regions could guide decision-making in some areas.

**Table:** 

The Norwegian Institute of Public Health started an action to support municipalities in the long-term goal—developing an intersectoral action plan to reduce inequalities in migrants' health at the local level. In this action, the NIPH mapped inter-sectoral activity on migration and health in municipalities based on data from municipal policy documents. They held meetings with municipalities to advocate for change on the issue and to over the long term include inter-sectoral action on migrant health in municipal policy and plans, to reduce inequalities in health at municipality level in Norway. The Directorate of Health and the participating municipalities have prioritized the continuation of this initiative to strengthen intersectoral action.

**Table:** 

The Escuela Andaluza de Salud Pública in collaboration with the Ministry of Health in Spain designed, implemented, and evaluated an online training course to tackle training needs of professionals in the health system working with migrants in Spain. The aim of the action was to address, with a systemic approach, the required skills for providing quality of healthcare to migrant population and effective welcoming recent migrants, with a special focus on social determinants of health and equity. The course provided participants with relevant JAHEE [HP-JA-2017] [801600] • 12 content on health in the context of migration, through a set of resources to incorporate new skills to address cultural diversity in their daily practice.

**Table:** 

Thanks to JAHEE, the Italian National Institute for Health, Migration and Poverty (INMP) developed a multilingual information tool (“La tua salute” mobile App) that is unprecedented in Italy and useful for fostering migrants’ awareness of their own right to health (even for those not entitled to NHS inscription) and their knowledge about NHS available services, women's and children's health, prevention (i.e. cancer screening, etc.) and healthy lifestyles at all ages. It is therefore an instrument to promote migrants health literacy and empowerment too. The App was developed with close attention to cultural issues related to health promotion and services utilization. The App’s effectiveness is monitored through the users rating tab and their answers are considered as their information needs for App updating.

**Table:** 

Through this action, Public Health Wales aimed to develop and disseminate health literacy resources in various languages. In response to COVID-19, the action was further expanded to produce additional information about accessing support during the pandemic, and information about mental health symptoms and signposting. Materials were provided in pictorial form with minimum text translated into over 20 languages, to address the inequality of access to healthcare information; hard copy versions were provided for those whose online access was restricted because of funds or equipment.

**Table:** 

As part of JAHEE, Portugal organised a training Plan and training sessions to support the training of health service providers, administrative personnel and social workers within the national health service primary health care structure on migration and health. An interactive e-learning course was specifically designed to address public health and migration, the psychosocial aspects of migration, intercultural mediation, and rights of access to healthcare in Portugal. Professionals were selected and invited by the Directorate-General of Health and the Regional Health to complete the e-learning course. Furthermore, the action will also directly contribute to national health plans and the overall approach towards migration.	

Thirteen countries participated in WP8-Improving Access to Health and Social Services for those Left Behind *(Bosnia and Herzegovina, Bulgaria, Cyprus, Czech Republic, France, Greece, Italy, Poland, Portugal, Romania, Serbia, Spain, Sweden)* and seventeen actions were implemented with the aim of reducing health inequalities and related social services in the participating countries. Three major categories of actions were identified: 1. operational interventions addressed to people in situation of vulnerability; 2. interventions aimed at building capacity for improving access and reducing inequities; 3. monitoring for contributing to equity accountability.

Shown in the box are some actions taken by the JAHEE partners to improve access to health and social service.

**Table:** 

Spain improved access to oral health services among disadvantaged children and youngsters in a comprehensive and equity-seeking manner by means of a cross-cutting action involving the whole of society in the Avilés Area. Implemented by the Ministry of Health of the Principality of Asturias (Asturias Region), in collaboration with the Health Area of Avilés, the specific activities included: (i) performing an oral examination and a first assessment of that child's cavity risk conditions, as part of the primary care pediatric services, (ii) educating children on how to achieve adequate oral health through Early Childhood Education (3–5 years) and Primary Education (6–12 years), as part of the project" La Conquista de la Boca Sana" and (iii) disseminating the project among those responsible for Social Welfare and Municipal Social Services.

**Table:** 

As part of JAHEE, France piloted community-based sexual health centers in cities with high prevalence of HIV and Sexually Transmitted Infections (STIs). The centers would offer a 'one-stop-shop' model providing a comprehensive response adapted to the specific health needs of vulnerable groups. Four sexual health centers (Paris, Lyon, Montpellier, and Marseille) were selected to participate in the pilot project, with the aim of increasing the number of entry points into a health pathway for vulnerable groups. The pilot project should lead to a decrease of at least 15% in the number of new HIV infections over the project period within the respective territories and have 100% of participants screened for hepatitis, 95% of participants caught up on their Hepatitis B vaccination and an overall reduced incidence of STIs.

**Table:** 

As part of JAHEE, Serbia organized home visits to potentially isolated older people by primary care practitioners. The aim of this action was to increase access to health care of older people with disabilities. Home treatment service teams visited the homes of a person with limited mobility to perform examinations and included them in a register of patients and their treatment plans. Forty-six examinations were carried out by a team of doctors and nurses at patients’ homes in five municipalities in the City of Nis. Those with recognized needs for home treatment were added to the group of patients that will continue to receive services offered by the Home Treatment Department.

**Table:** 

According to a Decision of the Romanian Parliament no. 39, milk formula will be provided free of charge for infants when mothers are not able to breastfeed, have a medical condition or have insufficient milk to support the development of the child. The National Institute of Mother and Child Health in Romania is by law responsible for JAHEE [HP-JA-2017] [801600] • 14 implementing provisions in the national health programme to prevent malnutrition in infants between 0–12 months. As part of JAHEE, they implemented an action to ensure that their services reach mothers in socially disadvantaged situations, to ensure that their babies receive a nutritional evaluation and that they are provided with free milk formula. On their online platform, they have also incorporated a special section dedicated to the activities for the milk formula distribution, that can also be used to monitor the action.

**Table:** 

As part of JAHEE, the government of Bulgaria included a provision in the National Programme for the Improvement of Maternal and Child Health (2021–2030) to improve access to services to uninsured pregnant women, many of whom are Roma. This is in response to analyses that have shown that current measures being taken for pregnant Roman and other uninsured women in the country are not sufficient. The goal is to make the new services sustainable for at least 10 years.	

Sixteen countries participated in WP9-Health and Equity in All Polices *(Belgium, Bulgaria, Croatia, Estonia, Finland, Greece, Italy, Lithuania, Netherlands, Poland, Portugal, Romania, Slovakia, Slovenia, Spain, United Kingdom)* and sixteen actions were implemented. The actions were collected in two main groups: 1. Assessments and reporting; and 2. Structure and mechanisms of governance.

Shown in the box are some actions taken by the JAHEE partners to improve the concept of Health and Equity in All Polices

**Table:** 

All public bodies in Wales are legally obliged to contribute to the delivery of the seven overarching goals of the groundbreaking Well-being of Future Generations Act (2015), which include ‘a healthier Wales’ and ‘a more equal Wales’. As part of JAHEE, Public Health Wales commissioned Kingston University London to undertake a literature review to assess what has been done during the first few years of implementation, to take forward the Act’s requirements and ambitions relating to governance and sustainable development, with examples drawn from government, regional and organisational levels. The research also drew on findings from around the world to identify how best to apply to implement the Sustainable Development Principle (the ‘five ways of working’) embodied in the Act. The findings from the Literature Review were used to underpin the development of the Health and Sustainability Hub tools and resources. The tools and resources were tested with over 200 colleagues across the NHS and in public bodies in Wales, and by JAHEE partners, who also found them relevant and applicable in a variety of cultural contexts, to implement the globally agreed Sustainable Development Goals and a reduction in health equity.

**Table:** 

In Italy an action was undertaken as part of JAHEE by the Istituto Superiore di Sanità (Italian National Institute of Health) and Piedmont RegionASLTO3 that aimed to support and coordinate centrally the introduction of the equity lens approach in the new National Prevention Plan and in the single Regional Prevention Plans (2020-2024). The Plan made Health Equity Audit a binding obligation; the action involved a capacity building process in all 20 Italian regions to help fulfil this new obligation. It demonstrated how the combination of a new legal duty and technical support for capacity building can help to mitigate the heterogeneous and fragmented capacity of policy response to inequalities in prevention.

**Table:** 

In the context of JAHEE, the Ministry of Health in Poland established a cross-cutting working team to analyze how policies in other areas impact on health inequalities. Its task was to work out the best approaches to shape and assess public policies that contribute to a reduction of health inequalities as set out in the Public Health Act. The working group assessed how it can engage with these sectors beyond the health sector, like social protection, housing, education and agriculture, as required by the Polish National Health Programme. It also identified how these initiatives impact on the health of vulnerable groups, and what can be done.

**Table:** 

As part of JAHEE, the National Center of Public Health and Analyses (NCPHA) in Bulgaria supported one Bulgarian municipality to develop an Action Plan on health equalities in all policies. They selected a municipality and established a multisectoral work group to develop an action plan for HEiAP at municipal level. They also undertook a situation analysis, including a stakeholder analysis, to outline local needs in relation to HI. Priorities were established on this basis, and activities planned, to support municipal plans and establish stronger cross sectoral planning and cooperation to in the long term improve the health and well-being of the local population.

**Table:** 

While there are several surveys to provide a partial view of health inequalities in Croatia, the ‘full’ picture, and what is being done by whom to address the situation, is incomplete. The National Institute of Public Health in Croatia undertook an extensive situation analysis to identify how HI are being addressed, who is responsible for doing what, and if there are mechanisms of intersectoral cooperation in place. As a result of this JAHEE activity, a Unit has been set up within the NIPH to coordinate this work.

**Table:** 

As part of JAHEE the Belgian Federal Public Service Health, Food Chain Safety and Environment, comprised of the departments of the Environment, Food, and Healthcare, mainstreamed awareness of health and environmental inequalities into the work of these departments. They did this by appointing ‘ambassadors’ who will serve as the single point of contact within the DG and/or service to sensitize colleagues about health inequalities and to deal with questions on this topic. They also initiated a reflection process whereby all officials that are preparing a new policy or initiative apply a checklist that will help them consider what can be done to minimize the negative and maximize the positive impacts on health inequalities, and how to ‘sell’ JAHEE [HP-JA-2017] [801600] • 8 the updated proposal to decision makers. The ambassadors will meet at least four times a year to exchange good practice and to support one another.

**Table:** 

As part of JAHEE, the National Institute of Public Health in Slovenia explored how they could use policy indicators as another approach to shed light on the causes of health inequalities. In other words, they explored policy developments between 2011 and 2021, and compared this with reports on levels of health inequalities in the country, to obtain a better understanding of how different policies can influence health equity. This approach enhanced networking among sectors, led to more aligned reporting and to a better understanding of the influences of policies on equity. Impact was assessed based on process. indicators (number of engaged sectors, number of meetings) and output indicators (Health Equity report for Slovenia, case study report) and on changes in the attitudes of health-related sectors towards this new approach.

**Table:** 

XarxaSalut, is a network of municipalities in Valencia, Spain that are committed to taking forward the local Health Plan that calls on HiAP. As part of JAHEE, with the support of the Conselleria de Sanitat Universal i Salut Pública, the network developed an equity lens in the form of a simplified screening tool that was adapted to and suitable for use at the local level. The resulting checklist, or tool, called Fem Salut?, or ‘Are we doing health?’ was piloted in six municipalities in Valencia, on a range of different policy and other initiatives. These included: healthy parks to promote physical activity amongst older people, sustainable mobility plans, and the recovery of places for young people to socialize. Amongst the ‘contextual’ adaptations made were: changes to the terminology to make it less technical and more understandable to citizens; the introduction of more qualitative methodologies; the introduction of more explicit supporting questions on issues that could affect equity, e.g., “Does this initiative reduce potential architectural barriers, and/or ensure access to elevators?”

**Table:** 

As part of JAHEE, the Federal Public Service Health, Food Chain Safety and Environment in Belgium launched a new program “One World, One Health” to promote actions concerning “Health and Environment in all policies”, with a focus on equity. The main objectives were (1) to develop and implement an impact assessment tool to measure the social impact of health and environment projects; (2) to raise awareness in the societal debate for the need of a proportional universalism approach in the implementation of health and environment policies. There are different cross-sectoral commissions at federal level in this country, and they will use these tools, and related working groups to raise awareness of the programme and its focus on equity.	

## Discussion

The JAHEE initiative, funded under the third EU Health Programme, represented an important opportunity for 24 European countries to work jointly to address health inequalities with concrete actions. JAHEE was the first opportunity where so many European countries agreed to improve their capacities of policy response addressing a few needs and priorities.

*Strengthening governance* for health equity under the HEiAP approach*,* including the non-health sectors, was recognized as a key to foster the integrations of health equity approach in national and local plans [12–17]. Furthermore, health equity lenses should be applied to facilitate the translation of this *HEiAP* approach into practice. This should go hand in hand with the data collection to support actions planning and understand their effectiveness. Each country is encouraged to develop a strong *health inequalities monitoring system* capable of collecting the evidence needed to support and encourage policy makers in developing effective responses to address health inequalities [18–22]. JAHEE WP5 showed that access to health data is normally the least challenging area, while the availability of a social stratifier in health data is still difficult. A country that wishes to improve its existing HIMS should consider prioritising communication along the development, implementation and evaluation stages to ensure active involvement of policymakers and other stakeholders. There are many potential target groups with an interest in HIMS, e.g. policy makers at all levels, health professionals, NGOs, media and the general public [23]. Moreover, JAHEE focused on *building healthy local communities and environments*. This local mission could be facilitated and assisted by regulation and capacity building from the national level. It is in the municipal setting that education throughout the life course is provided, as well as adequate conditions for housing and healthy habits, and regulation of urban development and of business or working conditions, creating many entry points for action [24–30]. *Improving universal access to health and social services* remains the other essential component for an adequate policy response to health inequalities. Reducing and overcoming barriers to access to health care with the aim of leaving no one behind is a fundamental mission of the health and social sector. Having the task of leading all other non-health sectors towards HEiAP, the health sector should demonstrate to be able to reduce inequalities in health services in the first place [31–34]. Finally, *migration and health* proved to be a priority area for two reasons. Any policy action and service that is responsive to the specific rights and needs of immigrants and minorities is also responsive to the needs and rights of the more socially disadvantaged groups. Moreover, the issue on migrant health and socio-economic differences needs better connections. Indeed, for some researchers, the health disadvantages experienced by immigrants mainly reflect their relatively weaker average socioeconomic position (SEP). Other researchers, however, believe that the migrant status has a direct effect on the health status or interact with the unequal health effect mediated by the SEP [35–41].

The JAHEE initiative has both strengths and limitations.

Among its greatest strengths, first the development of concrete actions aimed at improving the capacity to reduce health inequalities has shown that this can be done, even if it was only one step in a long-term journey with a long-term perspective. In this regard, it would be important for each country to periodically repeat the CAs, to understand the mechanisms of inequality and to identify gaps, needs, barriers and enabling factors for action. Besides, three main JAHEE legacies were produced: 1. a well established European community of practice for long-term future initiatives; 2. the road map for improving capacities in policy response; 3. the repository of promising practices designed to last beyond JAHEE.

One of the main limitations is related to the evaluation of the effectiveness and impact of JAHEE.

A formal evaluation of the impact of JAHEE would involve assessing quantitative changes in health inequalities over time attributable to the JAHEE’ actions. Nonetheless, this was not feasible within the scope of this JA due to the heterogeneity of the actions and the fact that such effects need to be addressed in a long-term perspective. However through JAHEE it has been possible for most of the countries involved to create a long-lasting impact on health inequalities. For most of the actions, outcomes are expected to be sustained beyond JAHEE, and for many actions outcomes contributed to the development or were integrated in national, regional, or local initiatives.

## Conclusion and recommendations

JAHEE represents a common effort to strengthen capacities to reduce health inequalities in 24 European countries and has provided evidence on what can be done and still remains to be done, to continue to “do more” and”do better” to improve capacities in policy responses to address health inequalities. At the beginning of the JA the participating countries were clustered according to a baseline assessment of health equity governance, in three main clusters: “do some”, “do more” and “do better”. The actions implemented during JAHEE showed that almost all countries now seem to be doing "something". All countries are ready to invest their efforts to move forward.

We encourage EU Institutions and EU MSs to prioritize the issue of health inequalities, through more explicit reference to the issue in their key policies and programmes, like plans to build a stronger European Health Union and in the EU Health4All Programme itself. It is necessary for public authorities to compel action through legislation whenever possible. It is also crucial to provide specific strategies and programmes with designated budgets, as well as clear guidelines on how public resources, like those of the EU Recovery and Resilience Funds, can be invested in ways that can contribute to greater health equity. Reducing health inequalities is a whole-of-society, multi-factorial challenge, but as JAHEE’s actions reflect, a great deal can be done in different contexts, by different levels of governance and in a range of areas, to make a difference. The greater the consistency of these efforts, and the more and better they are aligned, the stronger the impact. At EU level, the continuation of programmes like JAHEE is more essential than ever, to keep the issue on national agendas, and enable MSs to exchange knowledge, evidence, experiences and to strengthen capacities to reduce health inequalities. It is in this respect of value to invest in maintaining and developing the JAHEE community of practice, and the collective knowledge built, through a follow-up piece of work. This would enable further progress in efforts to mainstream a focus on health inequalities in all policies, as well as the recommendations outlined in this statement.

Another important consideration is that further studies/projects are needed to assess the impact of actions/interventions aimed at addressing health inequalities. JAHEE was not designed for this purpose, but primarily to create solid cooperation between EU countries and lay the foundations for future projects, which in the next years could be designed to put evidence-based policies into practice. As a final consideration it is worth to reflect the growing urgency to tackle equity issues in the new and unexpected war scenarios in Europe and worldwide. In this scenario, equity and health equity acquire a more prominent and central role in both research and practice and stress the need to urgently identify current and future health disparities also taking into account the social determinants of health of the new underserved populations and refugees.

At the end of JAHEE, on the basis of an analysis of the lessons learned from the progress made with implemented actions and from what remains to be done, a *Consensus Policy Document* including 10 final messages was elaborated and supported by the implementation of the 76 JAHEE actions:

1. “*Prioritize the reduction of health inequities, to “build back fairer*”. Health inequalities need to be prioritised in the political agenda in view of the post-pandemic recovery process. The health sector at all levels of governance, including the EU institutional level, needs to take the lead, to support more actions and investments needed to build capacity and translate political intentions into action to generate change.
2. “*Promote greater accountability*” through a specific body or group of actors to ensure that all sectors, both health and non-health, work together, according to the Health Equity in All Policies (HEiAP) approach, to implement multi-sectoral and level action to reduce health inequalities.
3. “*No data no progress.*” Robust Health Inequalities Monitoring System should be established to collect relevant and comparable data, stratified on a social covariate on health inequalities. This is essential to generate the evidence needed to raise awareness and engage policy makers to develop effective responses to tackle health inequalities and assess progress.
4. “*Apply an equity lens*” to all policy making processes, through instruments like health equity audits or health equity impact assessments, that can assess their differential impact across socio-economic groups and genders and bring a health equity perspective in all interventions/actions.
5. “*Mainstream migration into all aspects of health policy.*” It is crucial to ensure the inclusion of migrants into measures aimed at reducing inequalities for the general population and improving the access and quality of health and social services, which continue to be poorly implemented in many countries.
6. “*Improve access to service by reducing barriers and providing targeted interventions*”. Remove of all systemic barriers that prevent people from accessing adequate health care due to their social, economic, gender or cultural characteristics (including migrant status).
7. *“Strengthen municipal capacities”.* Strengthening local authorities who have direct access to local populations and the various actors that influence them, such as schools and businesses, and provide services of general interest. Local authorities can in fact influence many of the social, environmental and economic determinants of health that affect people's lives and health (e.g. community engagement, provision of social services, transport, provision of green spaces).
8. “*Ensure participation*”. Better participation and consultation can lead to a better understanding of the needs and interests of those concerned and implement more effective actions.
9. “*Invest in research, evaluation and exchange of what works*”. It is necessary not only to strengthen quantitative and qualitative research on the underlying causes of health inequalities but also to produce data on "what works" to reduce them. Particularly urgent is the need for a more systematic commitment in the evaluation of policies and initiatives, as well as in the cost–benefit and risk analysis of actions to improve health and reduce inequalities.
10. *“Seize the opportunities.*” More awareness should be raised on the need for a more integrated responsibility for health and a reduction in health inequalities, through the measures outlined above. EU MSs can seize the opportunities generated by EU policies and funds to respond to the COVID-19 pandemic, such as the EU Recovery and Resilience Funds and the EC Technical Support Instrument, and apply tools and resources available to focus attention on health equity.

These recommendations are addressed primarily to central governments and health authorities at EU, national and sub-national level. Building back fairer and building a resilient society has to be part of any national and global plan to respond to the threat and impacts of future pandemics and to address the chronic crises of climate change and environmental degradation. Achieving a healthier society is an indicator of a society's success, and achieving a healthier and fairer society is a key indicator of achieving a resilient and sustainable society.

## Data Availability

Non applicable.
